# Review of Short-Form Questions for the Evaluation of a Diet, Physical Activity, and Sedentary Behaviour Intervention in a Community Program Targeting Vulnerable Australian Children

**DOI:** 10.3390/children5070095

**Published:** 2018-07-13

**Authors:** Janelle A. Gifford, Josephine D. Gwynn, Louise L. Hardy, Nicole Turner, Lily C. Henderson, Christine Innes-Hughes, Victoria M. Flood

**Affiliations:** 1 Faculty of Health Sciences, The University of Sydney, 75 East St, Lidcombe, NSW 2141, Australia; josephine.gwynn@sydney.edu.au (J.D.G.); vicki.flood@sydney.edu.au (V.M.F.); 2 Charles Perkins Centre, The University of Sydney, Sydney, NSW 2005, Australia; 3 Sydney School of Public Health, The University of Sydney, Sydney, NSW 2006, Australia; louise.hardy@sydney.edu.au; 4 Centre for Rural and Remote Mental Health, The University of Newcastle, University Drive, Callaghan, NSW 2308, Australia; nicole.turner@sydney.edu.au; 5 Hunter New England Local Health District, Locked Bag 1, New Lambton, NSW 2305, Australia; 6 NSW Office of Preventive Health, Liverpool, NSW 1871, Australia; lilyh@heartfoundation.org.nz (L.C.H.); christine.inneshughes@health.nsw.gov.au (C.I.-H.); 7 Western Sydney Local Health District, Westmead, NSW 2145, Australia

**Keywords:** child, adolescent, Australian Aborigine, low-income populations, diet, physical activity, sedentary behaviour, questionnaires, obesity, community program

## Abstract

Childhood obesity is associated with low socioeconomic status in developed countries, and community programs can deliver cost-effective obesity interventions to vulnerable children and adolescents at scale. Evaluating these programs in a low-cost, time-efficient, and culturally appropriate way with valid and reliable measures is essential to determining their effectiveness. We aimed to identify existing valid and reliable short-form instruments (≤50 items for diet, ≤15 items for physical activity) suitable for the assessment of change in diet, physical activity, and sedentary behaviour in an Australian obesity intervention program for children and adolescents aged 7–13 years from low socioeconomic groups, with a focus on Aboriginal and Torres Strait Islander children. Relevant electronic databases were searched, with a focus on Australian literature. Validity and/or reliability studies using diet instruments (5), physical activity/sedentary behaviour instruments (12), and diet and physical activity/sedentary behaviour instruments used with Aboriginal and Torres Strait Islander (3) children were identified. Seven questions on diet, one question on physical activity, and no questions on sedentary behaviour were recommended. These questions can be used for evaluation in community-based obesity programs among Australian children and adolescents, including those from low socioeconomic groups and Aboriginal and Torres Strait Islander children.

## 1. Introduction

Obesity in childhood and adolescence is associated with low socioeconomic status in developed countries [1,2,3]. Children who are overweight or obese are more likely to experience health problems, including higher metabolic and cardiometabolic risk factors, asthma, negative psychological outcomes, poorer dental health, and sleep issues [4,5]. They also have a greater likelihood of being overweight or obese as adults [6,7]. Consequently, the lifetime societal and individual burden of childhood obesity may be substantial for those least able to bear the financial cost. Effective, cost-efficient obesity treatment and prevention programs that are accessible to vulnerable groups are critically needed.

Although economic evaluations of childhood obesity interventions have some limitations [8,9,10], there is evidence that childhood obesity interventions of even modest effectiveness are also economically viable from a broader policy perspective [8,11,12]. However, different types of interventions may vary in effectiveness depending on context. In their systematic review and meta-analysis of interventions to treat childhood obesity, Oude Luttikhuis et al. [13] found that specialised obesity clinics run within hospital settings were common among trials included in the review. Obesity clinics may be less available or accessible outside a research setting [13], and barriers such as transport may interfere with participation [14], particularly for non-metropolitan and socioeconomically disadvantaged children. In Australia, 27.4% of children and adolescents aged 5–17 were overweight or obese in 2014–2015 [15], with higher prevalence reported in Aboriginal and Torres Strait Islander children aged 2–14 years [16] and in children aged 2–17 from socioeconomically disadvantaged backgrounds [17]. It is therefore critical that programs that are more relevant for these groups be available. Community programs aiming to improve weight-related behaviours including diet, physical activity, and sedentary behaviours may be more accessible and have the potential for greater reach [13,18]. In a recent survey of obesity prevention practice in Australia, around two-thirds of community interventions targeted vulnerable groups such as low socioeconomic status, Aboriginal or Torres Strait Islander people, or culturally and linguistically diverse groups [19]. However, evaluation using instruments which demonstrate validity and reliability (while also being of low-cost and culturally appropriate) is needed to determine efficacy and support subsequent funding efforts. Moores et al. additionally report that collection of evaluation data may impact program engagement and attendance, and suggest that participant burden may be reduced and evaluation participation enhanced by implementing short instruments [20].

In Australia, Go4Fun^®^ is a government funded community-based healthy lifestyle program intended to improve the health, diet, fitness and self-esteem of children above a healthy weight aged 7–13 years and their families [21,22]. It is based on the UK MEND program (Mind, Exercise, Nutrition… Do it!) [23,24], and has been translated to a community-based program for the Australian context. In 2015–2016, the New South Wales Office of Preventive Health (OPH), which manages Go4Fun^®^, was undertaking quality improvement of the evaluation measures in the program and requested a rapid review of existing validated short-form survey instruments that assess diet, physical activity, and sedentary behaviour among children. The aim of this review was to inform the OPH of the best available evidence on, and recommend suitable evaluation questions in, the pre/post monitoring of programs that promote healthy eating and physical activity among children over the healthy weight range, such as Go4Fun^®^. Importantly, the review also considered suitability for Australian Aboriginal and Torres Strait Islander children and families.

## 2. Results

There were 18 unique validity and/or reliability studies meeting the inclusion criteria identified through two searches of the literature (see Section 3) and from experts in the fields: 5 short diet questions, 12 physical activity and/or sedentary behaviour measures, and 1 additional study identified from the search specific to Aboriginal and Torres Strait Islander children. Two of the papers identified in the search for non-indigenous specific studies were also identified in the search specific to Aboriginal and Torres Strait Islander children. A detailed narrative of each study included in the current review and the project-specific report is provided in Flood et al. [25]. A final list of the short-form questions recommended from the review can be found in the Supplementary Materials.

### 2.1. Diet Questionnaires

Five studies [26,27,28,29,30] were identified. Of these, one conducted reliability testing only [26], and the remaining studies conducted both validity and reliability testing [27,28,29,30]. Most of the studies did not indicate ethnicity and only half indicated weight status of the participants. Socioeconomic status was generally not indicated. The details of these studies are included in Table 1. 

The review identified questions on fruit and vegetable intake, water and sugary drink consumption, discretionary food intake, breakfast consumption, and eating in front of the television as common. The questions we recommended for use in evaluation included frequency of consumption of fruit, vegetables, sugar sweetened beverages, water, and discretionary foods [29]. These have been tested in Aboriginal and Torres Strait Island groups (see Section 2.3). An additional question on eating the dinner meal in front of the television [26] was also recommended.

### 2.2. Physical Activity and Sedentary Behaviour Questionnaires

Twelve validity and/or reliability studies on physical activity and/or sedentary behaviour measures were identified. Four studies were on physical activity only [31,32,33,34], four were on sedentary behaviour only [35,36,37,38], and four combined physical activity and sedentary behaviour measures [26,39,40,41]. There was a wide age range across the studies, and most of the studies did include details on ethnicity but not on the weight status of the participants. Only three studies involved parents answering the questionnaire [37,38,41]. Inclusion of children with low literacy was indicated in the pilot phase of one of the studies only [26], one included rural Aboriginal and Torres Strait Islander and non-Indigenous children [32], and three studies indicated mixed socioeconomic status (two used maternal education as an indicator) [33,37,41]. The details of these studies are included in Table 2.

Frequency and duration of different physical activity and sedentary behaviour domains were commonly reported. One question that had been tested for validity and reliability in a range of child/adolescent profiles [34] and used in the Australian setting [42] was recommended to evaluate physical activity. A suitable short-form question was not found for the evaluation of sedentary behaviour for the Go4Fun^®^ program, however a single question assessing children’s television screen time could be considered [26]. 

### 2.3. Diet and Physical Activity/Sedentary Behaviour Questionnaires in Aboriginal and Torres Strait Islander Groups

Only one dietary intake questionnaire [29] and two physical activity questionnaires [32,43] were identified that had been validated with Aboriginal and Torres Strait Islander children. There were no sedentary behaviour questionnaires identified. There was a narrow age range across the studies—none of the studies involved parental response, and only one study included the weight status of the participants [32]. Socioeconomic status and literacy levels were not indicated in these studies. The details of these studies are included in Table 3.

### 2.4. Quality Ratings

**Figure 1:**
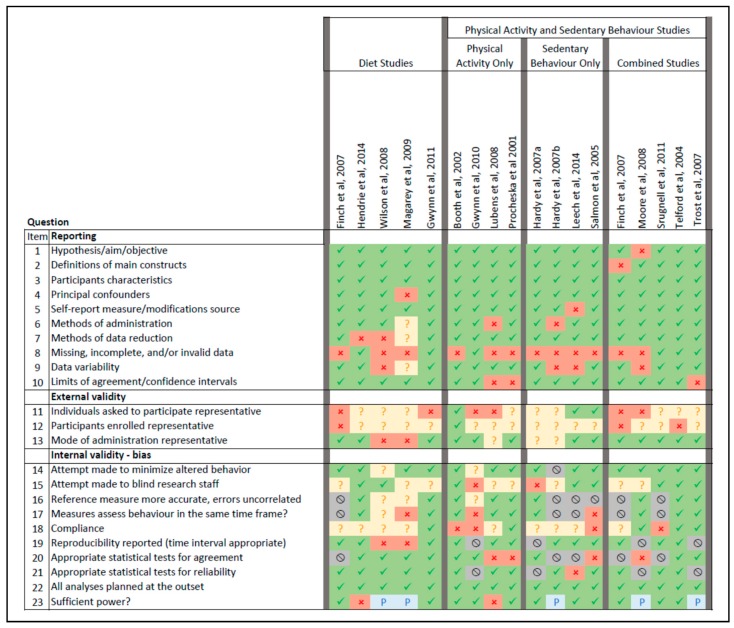
Visual summary of quality ratings for included individual studies. Green = yes, red = No, amber = unsure/unable to determine, grey = not applicable, blue = partially. The description of each question has been abbreviated.

Figure 1 summarises the quality rating for studies.

The reporting of studies was generally adequate, however many studies did not describe the characteristics of participants with missing, incomplete, and/or invalid data. The external validity of both diet and physical activity studies in terms of the representativeness of those invited to participate and those participating was often not able to be determined or was not adequate. However, the mode of administration of instruments was usually representative of similar study designs. Some aspects of internal validity such as attempts to minimise altered behaviour, appropriate statistics to test reliability (where applicable), planning of analyses, and having sufficient power were adequate across the studies. 

For validity studies, the reference measure was generally deemed to be more accurate than the test method and assessed behaviour in the same timeframe, however while studies on dietary measurement used appropriate statistics to assess agreement, this was not always the case for studies measuring physical activity/sedentary behaviour. For those studies that assessed reliability, statistics were mostly assessed to be appropriate. Clear exposition of compliance was frequently not provided, and blinding of research staff was either not able to be determined or not done.

## 3. Discussion

Valid and reliable short-form questions to measure dietary intake, physical activity, and sedentary behaviours are ideal for routine monitoring and evaluation of community programs to treat child and adolescent obesity. Despite the general availability of questionnaires, many of the articles reviewed in the current study did not have information on ethnicity, weight status, socioeconomic status, and literacy levels to determine their suitability for Go4Fun^®^ and similar programs, and many were not tested with a parent proxy. There were few studies specifically conducted with Australian Aboriginal and Torres Strait Island children. Although the general representativeness of the samples recruited across reviewed studies was not optimal, some recommendations were still able to be made based on question validity and reliability, suitability to address the targeted outcomes of Go4Fun^®^ and similar programs, and potential to be administered in different ways and among different population groups. Additionally, the quality of the studies from which questions were recommended [29,34] were satisfactorily rated by reviewers. 

The dietary factors evaluated by the recommended short-form dietary questions from the current review align with public health concerns and are similar to those identified by Golley et al. in their recent systematic literature review [44]. These questions demonstrated good reliability, however, satisfactory validity was not consistently demonstrated. Golley et al. similarly found that short-form food questions were more likely to be reliable than valid, and seldom both [44]. Responsiveness (ability to detect change) to an intervention compared with an alternative diet assessment at both time points was not identified in studies included in the current review. The recommended dietary intake questions may therefore be useful to indicate pre–post change in program interventions that target these behaviours, but not the magnitude of change. We found that frequency versus quantity of intake was generally found to be a superior measure. Children under 12 years old may be poor at conceptualising portion size even when prompts are provided [45]. Additionally, questionnaires with prespecified portion sizes may rely on serving sizes that more closely represent population food selection guides, however both adults and children may typically consume portion sizes that vary from guidelines [46,47]. 

The physical activity questions identified in the current review tended to perform poorly for validation of activity. Accurate assessment of activity in children is known to be difficult and may reflect the cognitive ability of this group in recalling different aspects of physical activity (e.g., intensity, frequency and duration) [48,49,50], as well as the sporadic nature of some activities, particularly of younger children [50,51,52]. In fact, in their systematic review and appraisal of studies of self-administered and proxy-reported physical activity questionnaires in youth, Chinapaw et al. determined that there were no questionnaires available with acceptable validity and reliability [53]. In any case, where program evaluation includes elements across the whole program, the inclusion of longer, more complex physical activity questionnaires [31,41] would impose an unacceptable burden, particularly for low literacy groups. Short-form questions which have demonstrated adequate reliability and validity in the USA as well as having been evaluated across different ethnicities [34] have been recommended for national monitoring in Australia [42], and one of these questions was recommended from our current review for evaluation of physical activity in community programs for children and adolescents. The value of using objective measures of physical activity (e.g., activity trackers such as pedometers and accelerometers) for children and adolescents is often discussed in the physical activity literature, however these may not be suitable. Gwynn et al. found that around 20% of children may remove these devices for various reasons [32]. If they are used in programs where assessment of change is important, it is recommended that a standardised protocol be used across timepoints [54].

Sedentary behaviour occurs across a range of activities in children and adolescents, for example inactive transport, desk-based schoolwork, and various forms of screen time. Australian national guidelines for children and adolescents aged 5–17 years recommend minimising the time spent on sedentary behaviour, and specifically limiting the use of electronic media (including television watching and computer use) to less than two hours a day [55]. The Adolescent Sedentary Activities Questionnaire (ASAQ) [36] was identified in the current review as having reliability and face validity, and does include questions on screen time; however, it is likely to be too lengthy for community program settings. We could not recommend any short-form questions to assess sedentary behaviour more broadly, however a single question assessing children’s television screen time [26] was considered to be potentially suitable for use in community programs if validated in the target population. Although a number of sedentary behaviours are associated with reduced energy expenditure and passive consumption of food [56], the most common measure of sedentary behaviour in this children and adolescents is television watching [57]. In their systematic review of over 200 studies, Tremblay et al. found that watching television for more than 2 h per day was associated with a range of adverse health outcomes, including unfavourable body composition, decreased physical fitness, and poorer scores on psychosocial and academic measures [57]. However, in community programs that target multiple forms of sedentary behaviour, a more global measure would be required. An additional consideration is that screen devices are constantly evolving [58,59], which may require modification of questions that address screen time behaviour.

Few studies were available that were specifically tested in Australian Aboriginal and Torres Strait Islander groups, representing a gap in the literature. In addition to the three studies identified by our review [29,32,43], Thurber et al. recently evaluated the relationship of screen time and dietary factors such as sugar-sweetened beverage and discretionary food intake reported by carers to body mass index trajectories in Aboriginal and Torres Strait Islander children [60]. However, the instrument used was not validated. Healthy physical activity and eating may be experienced differently among Australian indigenous children, as explained by Crowe et al. in their qualitative study of 40 Australian Aboriginal and Torres Strait Islander children aged 5–12 years recruited from the southeastern coast of Australia [61]. They found that healthy lifestyle behaviours were connected and influenced by cultural connections and activities [61], which may need further consideration in future questionnaires that measure diet, physical activity, and sedentary behaviours in this group. 

A strength of this study is the consideration of short-form questions suitable for vulnerable child and adolescent populations, including Australian Aboriginal and Torres Strait Island groups. Children from Aboriginal, Torres Strait Islander, and low socioeconomic groups have higher rates of obesity than in the general population [16,17] and there is a need for measures to evaluate suitable programs for these children. A further strength of this study is the inclusion of only short-form questions since many available questionnaires for use in research are lengthy, burdensome, and not suited to a community intervention setting. A limitation of the review is that psychometric assessment of some of the included questionnaires may have favoured recruitment of children from less disadvantaged backgrounds, and as such it may be less applicable for very disadvantaged children, however most of the dietary questions identified were tested for validity and reliability in “priority funded” (disadvantaged) schools. A further weakness of the current work is the timeframe of the review. The searches were completed in mid-2016 due to the requirements of the commissioning body.

## 4. Materials and Methods 

This review was conducted by a research team with expertise in: diet and physical activity interventions with Australian Aboriginal and Torres Strait Islander children as well as non-Indigenous children, the development and interpretation of relevant measurement instruments, and in associated validity/reliability studies.

### 4.1. Search Criteria

Searches were devised to locate: (1) validity/reliability studies on diet, physical activity, and sedentary behaviour measurement instruments suitable for Australian children and adolescents aged 7–13 years; and (2) studies with a focus on Australian Aboriginal and Torres Strait Islander children; hence, Australian papers were the focus of the search. The search strategy was developed by the review team, and one researcher (J.A.G.) conducted a systematic literature search to identify studies addressing the review questions. English language studies published between 1 January 2005 and 18 April 2016 were identified from the following electronic databases: Medline, CINAHL, EMBASE, and ATSIhealth. Search terms are shown in Table 4.

### 4.2. Selection and Inclusion Criteria

The literature search predominately comprised Australian studies in peer-reviewed journal articles, however selected publications from the international literature were also included if they met the inclusion criteria. Diet, physical activity, and sedentary behaviour questions were also sourced from the Parenting, Eating and Activity for Child Health [62] and GRx Active Families [63] as these were known childhood obesity programs in Australia and New Zealand respectively. 

Studies were included where the following items were described:

- components of diet, physical activity or sedentary behaviour questions relevant to current Australian nutrition and physical activity/sedentary behaviour policies for those aged 5–12 and 13–17 years, or that make a significant contribution to components of concern identified in policy documents; and
- short questionnaires with ≤50 items for diet [44] and ≤15 items for physical activity (expert opinion); and
- validity or reliability information in the population of interest (7–13 year old Australians); and
- questionnaire administration details indicating completion by children/adolescents or parent proxy.

**Figure 2:**
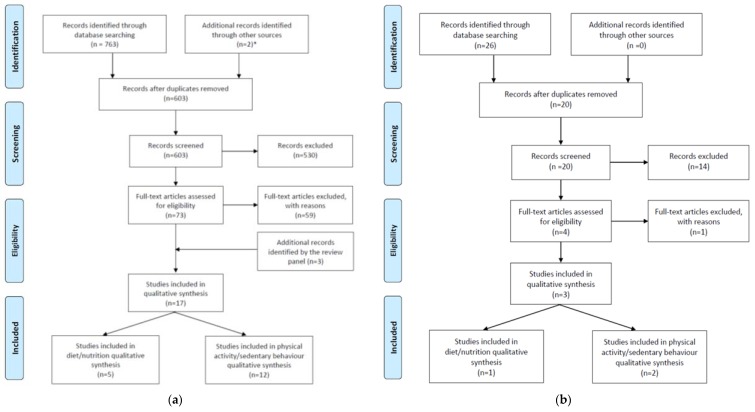
Flow of information through the different phases of the rapid review for identification of (**a**) studies on diet and physical activity/sedentary behaviour, and (**b**) studies on diet and physical activity/sedentary behaviour that specifically relate to Aboriginal and Torres Strait Islander children. The PRISMA (Preferred Reporting Items for Systematic Reviews and Meta-Analyses) diagrams are modified from Moher et al. [64]. * [62,63].

One reviewer (J.A.G.) screened the titles and abstracts of studies identified from the searches following removal of duplicates. Studies not meeting inclusion criteria were removed by the same reviewer. The same inclusion/exclusion criteria were applied to the full text of the remaining studies. Validity/reliability studies from the reference lists of relevant intervention studies and systematic reviews which met the inclusion criteria were included (if publication date was prior to 2005, inclusion was based on expert opinion). Additional references were included as advised by the review team. Figure 2 illustrates the flow of information through the different phases of the review according to the PRISMA (Preferred Reporting Items for Systematic Reviews and Meta-Analyses) process [64].

### 4.3. Data Extraction

Data were extracted by two researchers (J.A.G., J.D.G.) in standardised tables that included: author, year and country of study, program setting and name (if applicable), design, characteristics of the participants (sample size, ethnicity, age, sex, weight status, literacy details, comparison group), tool type and number of items, response variables, recall period, administration method, respondent (child or parent), respondent burden, duration of the study, period between administration (for reliability), reliability statistics, reference method (for validity), and validity statistics. 

### 4.4. Assessment of Quality

The methodological quality of each paper that met inclusion criteria was independently assessed by two of four members of the review team (J.A.G., J.D.G., L.L.H., V.M.F.) using a modified version of the Hagströmer–Bowles Physical Activity/Sedentary Behaviour Questionnaire Checklist (HBQC) [65], which is based on the scale devised by Downs and Black [66]. Members of the review team did not rate studies for which they were also an author.

The HBQC was modified to assess dietary validation/reliability papers simply by inserting relevant terminology in place of the physical activity/sedentary behaviour terminology. Additionally, in order to assess reliability in the studies, one question was added as follows:

“*Were the statistical tests used appropriate to assess reliability for the main physical activity constructs between tests for the self-report measure?*”

(The statistical techniques used must be appropriate to the data e.g., intra-class correlation co-efficient, weighted kappa).

While the HBQC scores items numerically, questions were simply assigned a value of yes, no, or unable to be determined/unsure for the current review. A “partially” option was available for the final question on statistical power, as per the HBQC. To accommodate studies that reported either validity or reliability but not both, a not applicable (N/A) option was included for relevant questions. A decision was made not to score the papers numerically because some individual questions may have more or less perceived importance qualitatively, and some methodological areas may have more or less questions; these two factors may bias the impression of the overall quality of the paper for low or high numerical scores. 

All four raters met to discuss the ratings and settle differences in ratings at an interim stage to assist with consistency by checking interpretation on quality items. When all papers had been rated individually, pairs met by phone or in person to discuss any differences. Differences that were not resolved by discussion were shared with the full team for adjudication. A record of decisions on interpretations was kept and shared with the team for review of past decisions to ensure all quality items were rated consistently within and across pairs of raters. 

### 4.5. Recommendations on Questions

Four members of the review team (J.A.G., J.D.G., L.L.H., V.M.F.) were involved in the final recommendations on the questions. Deliberations were made by group discussion following (and based on) data extraction of included studies. Factors considered by reviewers included the questions’ validity and reliability, suitability to address the objectives (targeted outcomes) of the Go4Fun^®^ program, and potential to be administered in different ways and among different population groups, in particular, Aboriginal and Torres Strait Islander children. Acceptable (statistically significant) validity and reliability were required. Specific outcomes measured in the Go4Fun^®^ program included daily servings of fruit, dairy foods, vegetables, sugar-sweetened beverages, and discretionary foods, as well as hours in physical activity and sedentary behaviour (screen time and non-active transport). 

## 5. Conclusions

In conclusion, there were some valid and reliable questionnaires that were considered useful for evaluation of our community-based obesity intervention targeting healthier diet, physical activity, and sedentary behaviour in Australian children and adolescents. Questionnaires selected for evaluation of programs need to capture the objectives of the intervention. The questions identified in this rapid review can provide information on the primary factors involved in child obesity prevention, that is, consumption of fruit and vegetables, sugar-sweetened beverages, and energy-dense nutrients, poor eating habits, time spent in physical activity, and screen time. Culturally appropriate support must be provided for Aboriginal and Torres Strait Islander children completing the survey questions. Central to this is ensuring a key role for their community members in survey administration and in interpretation of results. 

## Figures and Tables

**Table 1:** Diet questionnaires included in the review from peer-reviewed journal articles *.

Reference	Setting	Design	Sample	Questionnaire	Administration	Statistics
Finch et al., 2007 [26]	Local government primary school (Hunter Region, New South Wales (NSW), Australia).	Questionnaire development and reliability testing. Administered one week apart for reliability testing.	*n* = 245 children from Year 4 (*n* = 88), Year 5 (*n* = 84), Year 6 (*n* = 73). Mean age 10.7 ± 0.91 years. 52% F Ethnicity not indicated.	The School Food Eating Habits and Lifestyle Survey (SEHLS) with 35 items, including 27 on assessing “usual” food habits, five on “usual” physical activity and sedentary pursuits, and three on demographic variables.	Self-administered in class by children with teacher supervision. The questionnaire took around 30 min to complete in pilot testing.	**Reliability** Kappa 0.18–0.68 for food habit questions. All were within the 95% CI.
Gwynn et al., 2011 [29]	Many Rivers Diabetes Prevention Project. Eleven Department of Education and Training primary schools in three regional areas (north coast, NSW, Australia).	A short FFQ was completed twice, two weeks apart (reliability) and compared with the mean of three 24-h recalls (validity).	**Reliability** *n* = 241 age not specified. 59% F *n* = 92 Aboriginal and Torres Strait Islander, *n* = 149 non-Indigenous **Validity** *n* = 205 10–12 years, mean age 10.8 ± 0.7 years 58% F *n* = 78 Aboriginal and Torres Strait Islander children *n* = 127 non-Indigenous children	The Short Food Frequency Questionnaire (SFFQ) consisting of three demographic questions and 36 items (number of response categories 4–7) including 28 short questions on usual food intake.	Self-administered by the child. Culturally appropriate support was provided to Aboriginal and Torres Strait Islander children throughout the study.	**Reliability** Kappa 0.30–0.82. **Validity** 18 of 23 questions had increasing trends (*p* < 0.05) for mean daily weight and/or frequency as survey response categories increased.
Hendrie et al., 2014 [27]	Various, Adelaide (South Australia (SA), Australia).	The questionnaire was completed twice, one week apart (reliability). This was compared against the mean of three 24-h recalls (validity). Daily intake was used to calculate diet quality from both the questionnaire and the 24-h recalls.	*n* = 63 4–11 years, mean age 7.1 ± 2.1 years 97% F (parents), 44% F (children). 69.8% “normal” weight; 15.9% overweight/obese. Ethnicity not indicated. Generally from high-income, well-educated families.	The Short Food Survey (SFS) consisting of 38 items on “usual” intake, including 35 on food and three on beverages.	The survey was completed online by the parent.	**Reliability** The ICC was 0.43–0.94 for food groups/beverages, and 0.92 for the total diet index score (all *p* < 0.01). **Validity** The ICC was 0.04–0.44 for food groups/beverages and 0.44 for the total diet index score (*p* < 0.01). Percentage agreement across tertiles of index scores was 84% between the administrations and 43% when comparing the SFS with the mean of the recalls. Bias values were within the 95% CI.
Magarey et al., 2009 [28]	Five study samples of children from Adelaide (South Australia, Australia) and Sydney (NSW, Australia).	Reliability (studies 2 and 5; range 5–57 days between administration, median 10 days), internal consistency (Studies 1 (baseline), 3 and 5), and relative validity (Studies 4 and 5) using a 7-day food checklist, with the ability to detect change (Study 1).	*n* = 706 children (all five studies) age range 4–16 years Ethnicity not specified. **Study 1** *n* = 168 (baseline) *n* = 132 (at 6 months) Age 5–10 years BMI z score ≥1.07–4.0 (22% overweight, 78% obese) **Study 2** *n* = 39 Age 4–5 years 15% overweight, 7% obese **Study 3** *n* = 280 Age 4–5 years 15% overweight, 6% obese **Study 4** *n* = 126 Age 5–6 years **Study 5** *n* = 92 (reliability), *n* = 87 (validity) Age 5–16 years 16% overweight, 1% obese.	The Children’s Dietary Questionnaire (CDQ), a 28-item semi-quantitative FFQ. Four separate food group scores were calculated. Scores reflected food group intake in the previous 24 h by dividing items that measured intake in the past week by seven before summing.	Self-administered by the parent or caregiver (with or without researcher assistance). A trained researcher responded in three studies and a parent responded in two studies.	**Reliability** ICC 0.51–0.90 (*p* < 0.001, studies 2 and 5). **Validity** Pearson’s correlations 0.31–0.60 (*p* < 0.001, studies 4 and 5). **Internal consistency** Alpha co-efficient 0.13–0.76. Item: total correlation range from (0.10–0.37) to (0.49–0.62). **Ability to detect change** Significant changes in the expected direction for dietary patterns (baseline vs. 6 months).
Wilson et al., 2008 [30]	Eat well be active Community Programs, a community-based childhood obesity intervention project in South Australia. A mix of public and private, and metropolitan and rural primary schools.	Reliability (test–retest period not indicated/varied) and validity against 7-day food records (following both administrations of the questionnaire) was tested.	*n* = 134 (reliability), *n* = 117 (validity) 36% from Year 5, 33%, from Year 6, 31%, from Year 7 (not indicated which samples the proportions relate to). 10–12 years 62% F 66% attended metropolitan schools, 61% attended public schools. 14% overweight (9% M, 17% F), 6% obese (4% M, 8% F). Ethnicity not indicated.	The Child Nutrition Questionnaire (CNQ) assessing (a) dietary patterns relating to childhood obesity, and (b) behaviours, attitudes, environments and knowledge associated with healthy eating. 14 questions with a variable number of items; 12 scores were developed from the questionnaire and placed into five categories.	Self-administered by the child. Assistance was available. The questionnaire took 20 min to complete.	**Reliability** ICC 0.16–0.66. All were within 95% CI. **Validity** Spearman’s correlations 0.34–0.48 (all *p* < 0.01). Mean bias ranged from −1.2 to 0.6 and all values were within limits of agreement.

F = female, CI = confidence interval, FFQ = food frequency questionnaire, ICC = intra-class correlation co-efficient, BMI = body mass index, M = male. Articles are listed in alphabetical order. * Reproduced with minor modifications with permission from [25].

**Table 2:** Physical activity and sedentary behaviour questionnaires included in the review from peer-reviewed journal articles *.

Reference	Setting	Design	Sample	Questionnaire	Administration	Statistics
Booth et al., 2002 [31]	44 randomly selected high schools from three education sectors across NSW (Australia).	The questionnaire was administered twice, two weeks apart (reliability). It was tested against the Multistage Fitness Test (MFT; validity). The validity study was conducted independently of the reliability study (different students at different schools).	**Reliability** *n* = 226 (*n* = 121 Year 8, *n* = 105 Year 10). Mean age 13.7 ± 0.40 years (Year 8), 15.7 ± 0.40 years (Year 10) 48% F (Year 8), 29% F (Year 10) Ethnicity not indicated **Validity** *n* = 2026 (*n* = 1072 Year 8, *n* = 954 Year 10) Mean age 13.1 years (SD not given; Year 8), 15.1 years (SD not given; Year 10) 48% F (Year 8), 45% F (Year 10) 82% English-speaking, 7.0% Asian, 4.5% Middle Eastern, 4.2% European, 2.6% did not respond or were otherwise classified.	The Adolescent Physical Activity Recall Questionnaire (APARQ): four items with sub-items (a list of up to seven activities with frequency and time reported for each). The four items ask about organised and non-organised activities undertaken in summer (terms 1 and 4) and winter (terms 2 and 3).	Self-administered by the child.	**Reliability** Per cent agreement 67–83% and weighted kappa 0.33–0.71 for the three-category measure (vigorously active, moderately active, inactive). Per cent agreement 76–90% and kappa 0.25–0.74 for the two-category measure (adequately active, inactive). ICC (95% CI) for total energy expenditure from 0.30 (0.05–0.51) to 0.91 (0.82–0.96). **Validity** Higher mean laps in the moderately and vigorously active categories than the inactive category for girls, but only the vigorously active and inactive categories were different for boys (three-category measure). Higher mean laps in active vs. inactive category for all groups (two-category measure). Spearman’s correlations (energy expenditure and MFT laps): 0.14–0.39 (*p* < 0.01– *p* < 0.001).
Gwynn et al., 2010 [32]	Many Rivers Diabetes Prevention project. Eleven Department of Education and Training primary schools in three regional areas (north coast, NSW, Australia).	Validity was assessed against accelerometers for seven consecutive days.	*n* = 86 10–12 years; mean age 11.1 ± 0.7 years 59% F 23% overweight or obese *n* = 40 Aboriginal and Torres Strait Islander, *n* = 46 non-Indigenous children.	The Many Rivers Physical Activity Recall Questionnaire (MRPARQ; a modified version of the Adolescent Physical Activity Recall Questionnaire (APARQ)). All organised and non-organised physical activity in a “normal” week during summer and winter.	Self-administered by children seated in small groups with one or two members of the research team to assist, which always included an Aboriginal Health Worker for assistance.	**Validity** ICC 0.25 (*p* < 0.05) and Pearson’s correlation 0.37 (*p* < 0.05) for the overall average weekday daily MVPA accelerometer and MRPARQ.
Lubans et al., 2008 [33]	One secondary school in Oxford (United Kingdom (UK)) and one independent school in Newcastle (NSW, Australia).	Reliability was assessed in the UK sample via administration of the questionnaire twice, one week apart. Validity was assessed in the Australian sample by comparing the questionnaire data to accelerometer data from four consecutive school days (worn prior to questionnaire administration).	**Reliability** *n* = 87 Mean age 13.1 ± 0.9 years 44.8% F “Predominantly white” Mixed socioeconomic backgrounds **Validity** *n* = 51 Mean age 12.6 ± 0.5 years 47.1% F “Predominantly white” Mixed socioeconomic backgrounds.	Oxford Physical Activity Questionnaire (OPAQ); Eight items excluding demographics on the last seven days. Items include travel to/from school, activities at school, activities after school and on weekends, and other activities.	Self-administered by children. The questionnaire took 15 min to complete.	**Reliability** The ICC (95% CI) for moderate activity was 0.76 (0.63–0.84), vigorous activity 0.80 (0.70–0.87), and moderate to vigorous activity 0.91 (0.87–0.95). **Validity** Spearman’s correlations with moderate activity was r = 0.01 (NS), vigorous activity r = 0.33 (*p* = 0.01), moderate to vigorous activity r = 0.32 (*p* = 0.02).
Prochaska et al., 2001 [34]	Two high schools and two middle schools in San Diego, California, Pittsburgh (Pennsylvania, USA).	Three studies; two studies evaluated test–retest reliability and concurrent validity (against accelerometry) of six single-item and three composite measures of physical activity. A third study evaluated the best measure of those examined (and modified) in the previous two studies.	**Study 1** *n* = 250 Mean age 14.6 ± 1.4 years 56% F 36% white, 25% Asian/Pacific Islander, 17% African American, 9% Hispanic, 13% other. **Study 2** *n* = 57 Mean age 13.9 ± 1.7 years 37% white, 25% Asian/Pacific Islander, 4% African American, 12% Hispanic, 23% other. **Study 3** *n* = 148 Mean age 12.1 ± 0.9 years 65% F 27% white, 24% Asian/Pacific Islander, 7% African American, 5% Hispanic, 23% multiracial, 14% other.	The recommended measure had two recall assessing frequency of past seven days and “usual” activity performed for a total of at least 60 min per day.	Self-administered by children, supervised by research staff.	**Reliability** ICC 0.77 (kappa 61%). **Validity** MVPA correlation with accelerometer data r = 0.40 (*p* < 0.001).
Hardy et al., 2007a [35]	High schools near the study centre, Girls’ Healthy Development Study (Sydney, Australia).	Prospective cohort study (2.5 years), comprising five data collections, six months apart, between 2000 and 2002. Construct validity of the questionnaire was assessed using accelerometers worn at each time point for seven consecutive days.	*n* = 163 Mean ages for data collections 1 to 5 were 12.8, 13.4, 13.9, 14.4, and 14.9 years, respectively. 100% F ~25% non-English speaking background.	Sedentary Behaviour Questionnaire. Three main items (with sub-items) on sedentary behaviour on weekday and weekends and movie-going.	Self-administered by children.	**Validity** Bland–Altman plots showed <5% of data points were outside the limits of agreement (2 SD; 26.5 to 20.1 h/week).
Hardy et al., 2007b [36]	Four primary and four high schools randomly selected from Sydney (NSW, Australia).	The questionnaire was completed twice, two weeks apart (reliability) during autumn 2002.	*n* = 250 (Grade 6 = 98; Grade 8 = 73 and Grade 10 = 79) Mean age 11.3 years (Year 6), 13.3 years (Year 8) and 15.3 (Year 10). 44% F (overall), 49% F (Year 6), 47% F (Year 8), 37% F (Year 10). Ethnicity not indicated.	The Adolescent Sedentary Activities Questionnaire (ASAQ). Two main items with the same question; one on school days, one on weekends (11–12 identical sub-items except for the addition of church on weekends). “Usual” week during school term.	Self-administered by children.	**Reliability** ICC (95% CI) 0.01 (−0.88–0.46) to 0.95 (0.89–0.88). Most ICC ≥ 0.70. **Validity** Face validity was determined via pilot testing with a group of approximately 50 students (mean age 12 years).
Leech et al., 2014 [37]	Health Eating and Play study (HEAPS), state and Catholic primary schools in greater Melbourne (Victoria (VIC), Australia).	Cross-sectional study, including a 56-item FFQ, 7-day accelerometer data, and questions on sedentary behaviour. Questions were administered twice, 2–3 weeks apart.	*n* = 972 children (*n* = 362 5–6 years, *n* = 610 10–12 years). *n* = 133 parents (reliability study). 50% F 5–6 years, 56% F 10–12 years. 22% overweight/obese (5–6 years) and 29% overweight/obese (10–12 years) 19% maternal education low (5–6 years), 23% maternal education low (10–12 years) 92% of families of children aged 5–6 years usually spoke English at home, 87% of families of children aged 10–12 years usually spoke English at home.	Questions on sedentary behaviour asked about the number of hours (range: 0–6 or more hours), in 30-min blocks, their child watched (1) commercial and (2) non-commercial TV/DVDs on a typical school and weekend day. Usual daily TV viewing (minute/day) was calculated.	Self-administered by parents.	**Reliability** ICC (95% CI) 0.78 (0.69–0.84) usual daily TV viewing (minutes/day)
Salmon et al., 2005 [38]	Nineteen primary schools in Melbourne (VIC, Australia)	Parents completed a questionnaire about their child’s television viewing (validity). Questions were tested for reliability among a sample of the children (1 week apart) and parents (2 weeks apart).	*n* = 878 children with complete TV viewing data 54% F 22% F overweight, 5% F obese, 22% M overweight, 9% M obese 82% F (responding parents) Maternal education level was used as an indicator of SES; SES was evenly distributed across families (low SES, 30%; medium SES, 37%; high SES, 33%). **Reliability** *n* = 147 children Mean age 11.8 ± 0.8 years 55% F *n* = 156 parents mean age 40.0 ± 5.2 years 88% F 94% of all families reported speaking English at home, but it is not clear what the proportion was for the reproducibility element.	Three items on time spent in sedentary behaviour (watching TV, playing electronic games, and using the computer) were presented for a typical week (Monday to Friday) and a typical weekend (Saturday and Sunday).	Self-administered by children and parents.	Reliability * The ICC of the proxy-reported time (minutes per day) spent on each of these screen based behaviours ranged from 0.6 to 0.8. Validity * The ICC of the proxy-reported time (minutes per day) spent on each of these screen-based behaviours ranged from 0.44 to 0.61. * Report states that “Because proxy-reported sedentary time was more reliably reported, these items were used in analyses rather than the children’s self-reports.” (p. 1942).
Finch et al., 2007 [26]	One local government primary school (Hunter Region, NSW, Australia).	Questionnaire development and reliability testing. The questionnaire was administered twice, 1 week apart.	*n* = 245 (*n* = 88 Year 4, *n* = 84 Year 5, *n* = 73 Year 6) Mean age 10.7 ± 0.91 years 52% F Ethnicity not indicated.	School Food Eating Habits and Lifestyle Survey (SEHLS) with 35 items, including 27 on assessing “usual” food habits, 5 on “usual” physical activity and sedentary pursuits, and 3 on demographic variables.	Self-administered in class by children with teacher supervision.	**Reliability** Physical activity questions: kappa 0.57–0.71 Sedentary behaviour questions: kappa 0.51–0.59.
Moore et al., 2008 [39]	A local primary and secondary school, Northeast England (UK).	Children wore an accelerometer for 2 days (day 1, to desensitise them to wearing the monitor, and day 2, the day of recall) to assess validity of recalled activities.	*n* = 121 7–15 years, mean age 10.7 ± 2·2 years. 60% F 94% spoke English as their first language.	The Synchronised Nutrition and Activity Program^TM^ (SNAP^TM^) Recall of previous day activity. The overall number of items was not indicated. 29 common physical activities within the domains of sedentary activities, structured activities, household chores, play activities, and a free-text option were included. Transport activities were also assessed.	Self-administered by children (some availability of assistance was indicated, but this was not detailed). Web-based. The whole questionnaire (including nutrition questions) took 15–40 min dependent primarily on reading ability and Internet connection speed.	**Validity** Passing–Bablok regression equation established an overall bias of less than 4 min between the two methods, indicating good validity.
Strugnell et al., 2011 [40]	Three separate school samples from two Chinese weekend cultural schools from eastern metropolitan Melbourne (VIC, Australia).	Reliability of individual items and scales within the questionnaire determined by administration twice, 1 week apart.	*n* = 77 11–14 years, mean age 12 ± 0.8 years. 51% F 82% were of Chinese ethnicity (born in China, having both parents born in China, or having both maternal grandparents being born in China).	The Child and Adolescent Physical Activity and Nutrition Survey—Physical Activity (CAPANS-PA). The questionnaire the same as the Western Australian (WA) Child and Adolescent Physical Activity and Nutrition Survey (CAPANS) with minor modifications. Investigates 7 days of school and non-school based physical activity, sedentary behaviours, and associated correlates. Items within the CAPANS-PA were derived from several sources, including The Children’s Leisure Activity Study (CLASS) and APARQ.	Self-administered by children. Takes 15 min to complete.	**Reliability** Kappa (95% CI) for individual activities −0.04 (−0.07–0) to 0.82 (0.57–1.00). Kappa was >0.50 for most individual activities
Telford et al., 2004 [41]	Five state primary schools in Melbourne (VIC Australia).	Reliability of a parental proxy questionnaire and a children’s self-report questionnaire (2 weeks apart for parents and 1 week apart for children). Criterion validity assessed using accelerometry.	*n* = 169 children (*n* = 58 aged 5–6 years, *n* = 111 aged 10–12 years). *n* = 169 parents (*n* = 58 parents of children in the 5–6 year age group, *n* = 111 parents of children in the 10–12 year age group (2 excluded)). Mean age 5.3 ± 0.5 year (5–6 years), 37.4 ± 6.2 years (parents of children in the 5–6 year age group), 10.6 ± 0.8 years (10–12 year age group), 40.3 ± 5.9 years (parents of children in the 10–12 year age group). 37% F (5–6 years) 91% F (parents of children aged 5–6 years) 63% F (10–12 years) 83% F (parents of children aged 5–6 years) 77% of parents Australian-born (5–6 year age group). 75% of parents Australian-born (10–12 year age group).	The Children’s Leisure Activities Study Survey (CLASS) Consists of a list of 30 physical activities. Participants indicate participation in activities during a typical week (Monday to Friday) and during a typical weekend (Saturday and Sunday). For each activity, frequency and the total time spent is reported.	Self-administered by parents (proxy report for children aged both 5–6-years and 10–12-years), and children aged 10–12 years who completed the questionnaire in class guided by an investigator. The questionnaire took 10 min for parents to complete and 15 min for children to complete.	**Reliability** ICC for 10–12 years only: For self-report it ranged from 0.36 (*p* < 0.001) for total activity (frequency) to 0.74 (*p* < 0.001) for total activity (duration). For proxy report it ranged from 0.24 (NS) for total activity (duration) to 0.75 (*p* < 0.001) for vigorous activity (frequency). **Validity** Spearmans correlations between children (10–12 years) and proxy report: Vigorous activity: frequency rs = 0.13 (NS), duration rs = 0.19 (*p* < 0.05). Moderate activity: frequency rs = 0.07 (NS), duration rs = 0.14 (NS). Total activity frequency: rs = 0.25 (*p* < 0.01).

F = female, SD = standard deviation, ICC = intra-class correlation co-efficient, CI = confidence interval, MVPA = moderate and vigorous physical activity, NS = not significant, SES = socioeconomic status, M = male. Articles are listed in alphabetical order in the following sequence: physical activity, sedentary behaviour, combined physical activity and sedentary behaviour. * Reproduced with minor modifications with permission from [25].

**Table 3:** Diet, physical activity and sedentary behaviour questionnaires used in Aboriginal and Torres Strait Islander children included in the review from peer-reviewed journal articles *.

Reference	Setting	Design	Sample	Questionnaire	Administration	Statistics
Gwynn et al., 2011 [29]	Many Rivers Diabetes Prevention Project. Eleven Department of Education and Training primary “priority funded” (disadvantaged) schools in three regional areas (north coast, NSW, Australia).	A short FFQ was completed twice, two weeks apart (reliability) and compared with the mean of three 24 h recalls (validity).	**Reliability** *n* = 241 age not specified. 59% F *n* = 92 Aboriginal and Torres Strait Islander, *n* = 149 non-Indigenous. **Validity** *n* = 205 10–12 years, mean age 10.8 ± 0.7 years. 58% F *n* = 78 Aboriginal and Torres Strait Islander children, *n* = 127 non-Indigenous children.	The Short Food Frequency Questionnaire (SFFQ) consisted of three demographic questions, 36 items (number of response categories 4–7) including 28 short questions on usual food intake.	Self-administered by the child. Culturally appropriate support was provided to Aboriginal and Torres Strait Islander children throughout the study.	**Reliability** Kappa 0.28–0.89 in Aboriginal and Torres-Strait Islander children. Kappa 0.33–0.77 in non-Indigenous children. **Validity** 18 of 23 questions had increasing trends (*p* < 0.05) for mean daily weight and/or frequency as survey response categories increased.
Gwynn et al., 2010 [32]	Many Rivers Diabetes Prevention project. Eleven Department of Education and Training primary “priority funded” (disadvantaged) schools in three regional areas (north coast, NSW, Australia).	Validity was assessed against accelerometers for seven consecutive days.	*n* = 86 10–12 years; mean age 11.1 ± 0.7 years. 59% F 23% overweight or obese *n* = 40 Aboriginal and Torres Strait Islander, *n* = 46 non-Indigenous children	The Many Rivers Physical Activity Recall Questionnaire (MRPARQ), a modified version of the Adolescent Physical Activity Recall Questionnaire (APARQ)). All organised and non-organised physical in a “normal” week during summer and winter.	Self-administered by children seated in small groups with one or two members of the research team to assist, which always included an Aboriginal Health Worker for assistance.	**Validity** ICC 0.16 (*p* < 0.05) and Pearson’s correlation 0.31 (NS) for average weekday daily MVPA accelerometer and MRPARQ in Aboriginal and Torres Strait Islander children. ICC 0.31 (*p* < 0.05) and Pearson’s correlation 0.38 (*p* < 0.05) for average weekday daily MVPA accelerometer and MRPARQ in non-Indigenous children.
Trost et al., 2007 [43]	Public secondary schools from Brisbane South (QID, Australia).	Validity was assessed against a pedometer worn on the day previous to answering the questionnaire.	*n* = 122 13.8 ± 1.2 years 53% F *n* = 63 Aboriginal and Torres Strait Islander, *n* = 59 non-indigenous	24-h physical activity recall (the PDPAR-24). Participants entered the main activity (of 69) in which he/she participated during each 30-min time period between 9 a.m. and 9 a.m. in the previous 24 h (excluding midnight–5 am).	Children self-administered the instrument in groups of approximately five individuals under the supervision of the research team who followed a standardised administrator script.	**Validity** Spearman’s correlations for mean METs, vigorous physical activity, MVPA, and screen-based activity were 0.34 (*p* < 0.05), 0.34 (*p* < 0.05), 0.28 (*p* < 0.05), and −0.13 (NS), respectively, in Aboriginal and Torres Strait Islander children and 0.32 (*p* < 0.05), 0.26 (*p* < 0.05), 0.28 (*p* < 0.05), and −0.20 (NS), respectively in non-Indigenous children.

FFQ = food frequency questionnaire, F = female, ICC = intra-class correlation co-efficient, MVPA = moderate and vigorous physical activity, METs = metabolic equivalent of tasks, NS = not significant. Articles are listed in alphabetical order in the following sequence: the article on the diet questionnaire, the article on the physical activity questionnaire, the article on the combined physical activity and sedentary behaviour questionnaire. * Reproduced with minor modifications with permission from [25].

**Table 4:** Search terms *.

Field 1	Field 2	Field 3	Field 4	Field 5 ^†^
(diet OR diet * OR food) OR (“physical activity” OR exercise OR sedentary OR inactivity)	Child * OR teen * OR adolescen *	Survey OR FFQ OR food frequency questionnaire OR questionnaire OR screening OR checklist OR diet quality OR diet index OR physical activity index	Valid * OR reprod * OR reliab *	Austral * ((Aborigin * OR Torres Strait Islander OR Indigenous) AND Austral *)

^†^ For the general search, “Austral *” was used for Field 5, for the search focusing on Aboriginal and Torres Strait Islander children and adolescents, Indigenous-specific terms were utilised for Field 5. * Reproduced with minor modifications with permission from [25].

## Supplementary Materials

The following are available online at http://www.mdpi.com/2227-9067/5/7/95/s1.
