# Supplementary material for: Review of Short-Form Questions for the Evaluation of a Diet, Physical Activity, and Sedentary Behaviour Intervention in a Community Program Targeting Vulnerable Australian Children
Source: Children (Basel). 2018 Jul 13;5(7):95. doi: 10.3390/children5070095 (PMC6069381; doi:10.3390/children5070095)
Supplement: Supplementary file 1 [file children-05-00095-s001.pdf]

Supplementary Material: Recommended short-form questions

It is recommended that the parent complete the questions as a proxy if the child is less than 10 years old, and that the child complete the questions if they are more than 12 years old. Either the child or the parent proxy could complete the questions if the child is 10-12 years old. The questionnaire should indicate if the child or the parent completed the questions.

Recommended dietary intake questions

The following questions are taken from the Many Rivers Diabetes Prevention Project [1]. It is recommended that the parent complete the questions as a proxy if the child is less than 10 years old, and that good visual prompts accompany the questions; for example those used in the Students as Lifestyle Activists (SALSA) high school community intervention currently run in high schools in Western Sydney [2,3]

1. **How many serves of fruit do you USUALLY eat each day? (A 'serve' is 1 medium piece or 2 small pieces of fruit or a cup of diced pieces.) This includes all fresh, dried, frozen, and tinned fruit**.

    ☐ I don't eat fruit
    ☐ 1 serve or less
    ☐ 2 serves
    ☐ 3 serves
    ☐ 4 serves or more

2. **How many serves of vegetables do you USUALLY eat each day? (A 'serve' is half a cup of cooked vegetables or 1 cup of salad vegetables.) This includes all fresh, dried, frozen and tinned vegetables.**

    ☐ I don't eat vegetables
    ☐ 1 serve or less
    ☐ 2 serves
    ☐ 3 serves
    ☐ 4 serves
    ☐ 5 serves or more

3. **How much soft drink, cordials or sports drinks do you USUALLY drink (e.g. cordial, Coke, Lemonade, Gatorade)? (one can of soft drink = 1 ½ cups)**

- ☐ I don't drink soft drink
- ☐ Less than one cup a week
- ☐ About 1-3 cups a week
- ☐ About 4-6 cups a week
- ☐ About 1-2 cups a day
- ☐ About 2-3 cups a day
- ☐ 3 cups or more a day

4. **How much water do you USUALLY drink each day? This can be plain tap water or bottled water. (1 average bottle = 2 cups)**

- ☐ I don't drink water
- ☐ Less than one cup a day
- ☐ About 1 to 2 cups a day
- ☐ About 2 to 3 cups a day
- ☐ About 3 to 4 cups a day
- ☐ About 4 cups or more a day

5. **How often do you eat hot chips, wedges, or hot French fried potatoes?**

- ☐ Never or rarely
- ☐ Less than once a week
- ☐ About 1 to 2 times a week
- ☐ About 3 to 4 times a week
- ☐ About 5 to 6 times a week
- ☐ About once a day
- ☐ 2 or more times a day

6. **How often do you have meals or snacks, such as burgers, pizza, chicken, or chips from places like McDonalds, Hungry Jacks, Pizza Hut, KFC, Red Rooster or local take-away food places?**

- ☐ Never or rarely
- ☐ Less than once a week
- ☐ About 1 to 2 times a week
- ☐ About 3 to 4 times a week
- ☐ About 5 to 6 times a week
- ☐ About once a day
- ☐ 2 or more times a day

We also recommend the inclusion of a question on eating dinner in front of television [4] because eating meals in front of the television is associated with increased weight and poorer diet quality [5,6].

7. **How many times a week do you usually eat your meal at night in front of the television (TV)?**

   _______days a week

   OR

   ☐ I rarely / never eat in front of the TV

Recommended physical activity question

The following question is taken from Active Healthy Kids Australia [7], which is modified from Prochaska et al [8].

Over the past 7days, on how many days were you/your child engaged in moderate to vigorous physical activity for at least 60 minutes (this can be accumulated over the entire day, for example in bouts of 10 minutes) each day?

Moderate to vigorous activity is any activity that increases the heart rate and gets you out of breath some of the time

○ No days ○ 1 day ○ 2 days ○ 3 days ○ 4 days ○ 5 days ○ 6 days ○ 7 days

Suggested sedentary behaviour question

The following short question [4] may address the sedentary behaviour, however it is limited to weekdays and the screen time is limited to television viewing:

On weekdays how much time do you usually spend watching television (TV)?

_____ hours ________minutes

References

1. Gwynn, J.D.; Flood, V.M.; D'Este, C.A.; Attia, J.R.; Turner, N.; Cochrane, J.; Wiggers, J.H.; on behalf of the Many Rivers Diabetes Prevention Project Study Team. The reliability and validity of a short FFQ among Australian Aboriginal and Torres Strait Islander and non-Indigenous rural children. *Public Health Nutr.* **2011**, *14*, 388-401; DOI: 10.1017/s1368980010001928.
2. Shah, S.; Patching van der Sluijs, C.; Lagleva, M.; Pesle, A.; Lim, K.S.; Bittar, H.; Dibley, M. A partnership for health - working with schools to promote healthy lifestyle. *Aust. Fam. Physician* **2011**, *40*, 1011-1013;
3. Shah, N. *Summary Report: Impact of the SALSA Program in High Schools in Western Sydney 2013-2016*; Western Sydney Local Health District: Westmead, 2016.
4. Finch, M.; Begley, A.; Sutherland, R.; Harrison, M.; Collins, C. Development and reproducibility of a tool to assess school food-purchasing practices and lifestyle habits of Australian primary school-aged children. *Nutr. Diet.* **2007**, *64*, 86-92;
5. Vik, F.N.; Bjornara, H.B.; Overby, N.C.; Lien, N.; Androutsos, O.; Maes, L.; Jan, N.; Kovacs, E.; Moreno, L.A.; Dossegger, A., *et al.* Associations between eating meals, watching TV while eating meals and weight status among children, ages 10-12 years in eight European countries: The energy cross-sectional study. *Int. J. Behav. Nutr. Phys. Act.* **2013**, *10*, 58; DOI: 10.1186/1479-5868-10-58.
6. MacFarlane, A.; Cleland, V.; Crawford, D.; Campbell, K.; Timperio, A. Longitudinal examination of the family food environment and weight status among children. *Int. J. Pediatr. Obes.* **2009**, *4*, 343-352; DOI: 10.3109/17477160902846211.
7. Active Healthy Kids Australia. *Is Sport Enough? The 2014 Active Healthy Kids Australia Report Card on Physical Activity for Children and Young People*; Adelaide, 2014.
8. Prochaska, J.J.; Sallis, J.F.; Long, B. A physical activity screening measure for use with adolescents in primary care. *Arch. Pediatr. Adolesc. Med.* **2001**, *155*, 554-559.
